# Glyoxalase 1 Expression as a Novel Diagnostic Marker of High-Grade Prostatic Intraepithelial Neoplasia in Prostate Cancer

**DOI:** 10.3390/cancers13143608

**Published:** 2021-07-19

**Authors:** Liliana Rounds, Ray B. Nagle, Andrea Muranyi, Jana Jandova, Scott Gill, Elizabeth Vela, Georg T. Wondrak

**Affiliations:** 1 Department of Pharmacology and Toxicology, College of Pharmacy & UA Cancer Center, University of Arizona, Tucson, AZ 85724, USA; lrounds@email.arizona.edu (L.R.); jjandova@arizona.edu (J.J.); 2 Roche Diagnostics Solutions, Tucson, AZ 85755, USA; andrea.muranyi@roche.com (A.M.); scott.gill@roche.com (S.G.); liz.vela@roche.com (E.V.); 3 Department of Pathology, University of Arizona, Tucson, AZ 85724, USA; rnagle@email.arizona.edu

**Keywords:** prostate cancer patients, high-grade prostatic intraepithelial neoplasia, precancerous lesions, glyoxalase 1, immunohistochemistry, diagnostic marker

## Abstract

**Simple Summary:**

Prostate cancer (PCa) is the most commonly diagnosed cancer and the second leading cause of cancer-associated deaths in men in the USA. Glyoxalase 1 (GLO1) is an enzyme involved in energy metabolism in various tumor types including PCa. However, GLO1 expression has not been explored in the context of PCa progression with a focus on high-grade prostatic intraepithelial neoplasia (HGPIN), a frequent precursor to invasive cancer. Here, we have evaluated GLO1 expression by immunohistochemistry in tumor samples from a PCa patient cohort. Immunohistochemical analysis indicated that GLO1 is upregulated during tumor progression, observable in HGPIN and PCa as compared to normal prostatic tissue. Remarkably, GLO1 upregulation was identified as a hallmark of HGPIN lesions, displaying the highest staining intensity in all clinical patient specimens. Since current pathological assessment of HGPIN status solely depends on morphological features, GLO1 may serve as a novel diagnostic marker that identifies these precancerous lesions.

**Abstract:**

Glyoxalase 1 (GLO1) is an enzyme involved in the detoxification of methylglyoxal (MG), a reactive oncometabolite formed in the context of energy metabolism as a result of high glycolytic flux. Prior clinical evidence has documented GLO1 upregulation in various tumor types including prostate cancer (PCa). However, GLO1 expression has not been explored in the context of PCa progression with a focus on high-grade prostatic intraepithelial neoplasia (HGPIN), a frequent precursor to invasive cancer. Here, we have evaluated GLO1 expression by immunohistochemistry in archival tumor samples from 187 PCa patients (stage 2 and 3). Immunohistochemical analysis revealed GLO1 upregulation during tumor progression, observable in HGPIN and PCa versus normal prostatic tissue. GLO1 upregulation was identified as a novel hallmark of HGPIN lesions, displaying the highest staining intensity in all clinical patient specimens. GLO1 expression correlated with intermediate–high risk Gleason grade but not with patient age, biochemical recurrence, or pathological stage. Our data identify upregulated GLO1 expression as a molecular hallmark of HGPIN lesions detectable by immunohistochemical analysis. Since current pathological assessment of HGPIN status solely depends on morphological features, GLO1 may serve as a novel diagnostic marker that identifies this precancerous lesion.

## 1. Introduction

Prostate cancer (PCa) is the most commonly diagnosed cancer and the second leading cause of cancer-associated deaths in men in the USA. It is estimated that in the year of 2021, a total of 248,530 new cases will be diagnosed and 34,130 of those men will succumb to the disease [1]. The etiology of PCa is not well understood, although some risk factors including inflammation, metabolic factors, hormonal influences and genetic variation [2] have been associated with it. Prostatic intraepithelial neoplasia (PIN) is the only widely accepted histological condition preceding PCa, although it is unclear whether PIN arises from normal prostatic tissue or dysplastic epithelial cells [3,4,5,6]; also, high-grade PIN lesions (HGPIN) are now considered the most likely precursor of invasive PCa [7,8].

PCa is a disease with a disproportionate burden afflicting the elderly, and risk prediction is crucial. Most patients present with low-risk, relatively indolent tumors; however, 20–30% of patients present with tumor characteristics associated with high-risk PCa, more likely to progress and relapse [9]. In this group of men, early-stage intervention may limit the development of prostate cancer, and identification of this subpopulation of patients is critical for optimal therapy. Therefore, a lack of molecular markers with clinical utility for early detection and prediction of tumor progression represents an unmet medical need. Hence, effective intervention requires identification and development of improved diagnostic assays allowing early detection of disease progression.

Standard diagnostic immunohistochemistry of PCa currently involves detection of a-methylacyl-CoA racemase (AMACR; also referred to racemase) together with basal cell markers including p63 and high-molecular-weight cytokeratin (34βE12, HMWCK). However, the diagnostic value and correct interpretation of these markers require a distinct assessment of their morphological context [10]. For example, racemase overexpression is a molecular hallmark already detectable in HGPIN, thereby limiting the value of this marker to discriminate between precursor and advanced PCa [11,12]. There is continuous clinical interest in identification of additional markers that may prove helpful where AMACR staining is insufficient or ambiguous including fatty acid synthase (FAS), Golgi membrane protein 1 (GOLM1, also known as GOLPH2 or GP73), and ETS-related gene (ERG), useful in the context of transmembrane protease, serine 2 (TMPRSS2)-ERG gene translocation [10].

High glycolytic activity is an oncometabolic hallmark of cancer cells [13,14,15]. Indeed, dysregulated energy metabolism is an important driver in oncogenesis. Cancer cells turn to aerobic glycolysis for energy production in a process referred to as ‘the Warburg effect’ leading to accumulation of methylglyoxal (MG), a cytotoxic glycolytic byproduct that causes adduction of macromolecules by formation of advanced glycation end-products (AGEs). Glyoxalase 1 (GLO1), also known as lactoylglutathione lyase (EC: 4.4.1.5), is part of the glyoxalase system, playing a crucial role in cellular detoxification of spontaneously formed MG. In a two-step reaction, GLO1 first catalyzes the conversion of highly reactive MG to S-D-lactoylgluthatione. The resulting thioester, in the case of MG, is then hydrolyzed by GLO2 to produce D-lactate and reformed glutathione (GSH) [16]. The cytoprotective glyoxalase system prevents formation of AGEs and promotes cell survival. GLO1 upregulation has been described in the context of metabolic and inflammatory stress in vitro and in vivo [17,18,19,20], serving as a cellular mechanism to detoxify high levels of MG, particularly in cancer, where high glycolytic rates as a result of the Warburg effect are observed. Previous reports have shown upregulation of GLO1 in various cancers including melanoma [21], breast cancer [22,23,24], prostate cancer [25,26], gastric cancer [27,28], pancreatic cancer [29] and renal carcinoma (clear cell carcinoma) [30].

Interestingly, previous studies have reported GLO1 upregulation in PCa patient tissue samples detecting a wide range of staining intensity [25,26]. GLO1 displayed a strong association with Gleason grade, pathological tumor stage, and early biochemical recurrence (BCR). In addition, a positive correlation between circulating plasma GLO1 and TGFβ levels was identified in metastatic PCa patients, suggestive of a role of GLO1 in TGFβ-driven EMT was also confirmed by cell-based mechanistic studies [31]. Moreover, GLO1 upregulation appears to be a feature of prostate cancers characterized by *ERG* fusion and *PTEN* deletion [25,26]. In spite of significant variability in GLO1 prevalence in PCa specimens, these prior studies strongly indicate that GLO1 upregulation is associated with progression and aggressiveness.

Given the critical role of GLO1 as an enabler of disease progression via MG detoxification, it is important to further investigate and provide more evidence on the status of this marker, especially in PCa, where the mechanism of disease progression and aggressiveness is still poorly understood. In this study, we have evaluated GLO1 expression by immunohistochemistry in a tissue microarray cohort consisting of 882 prostate cancer specimens obtained from 187 cases with associated clinical data. GLO1 expression was identified as a distinct molecular marker characteristic of early PCa development as substantiated by high expression levels observed in HGPIN for the first time. 

## 2. Materials and Methods

### 2.1. Patients

Prostate specimens from 200 patients undergoing radical prostatectomies between the years of 1995 and 2007 at the University of Arizona Medical Center (UMC) and the Tucson Medical Center (TMC) were available at the Tissue Acquisition and Cellular/Molecular Analysis Shared Resource (TACMASR) at the University of Arizona Cancer Center, University of Arizona, Tucson, AZ, USA (Table 1).

The clinical database associated with this tissue cohort was originally generated from information obtained from de-identified pathology reports. Histopathological data included patient age, pre- and postoperative prostate-specific antigen (PSA), Gleason assessment at biopsy, and pathologic stage (T2 = organ confined tumor; T3 = capsular penetrating tumor). Gleason score and grade, as defined by the International Society of Urological Pathology [32], were revalidated and used for data analysis. Biochemical recurrence (BCR) was defined as postoperative PSA ≥ 0.2 ng/mL [33]. Specifically, upon resection, whole-prostate specimens were placed on ice and transported to the TACMASR facility, where they were dissected, sliced at 10 mm intervals, and placed in 10% neutral buffered formalin for at least 24 h. Prostate specimens were finally sectioned at (5 mm), processed and embedded to produce 20–50 formalin fixed paraffin embedded (FFPE) blocks per specimen. An H&E section per block was evaluated by a board-certified pathologist to generate maps of the tumor-containing areas. Multiple cores (3 mm) from selected tumor areas and adjacent normal tissue for each prostate specimen were used to assemble a total of 17 tissue microarrays (TMAs). Each TMA was composed of 59 randomly assigned specimen cores and one orientation tissue from a normal organ different from prostate. All steps during the pre-analytical phase including sample preparation and processing were monitored carefully to preserve tissue integrity and avoid degradation of tissue biomarkers. This study was reviewed by the University of Arizona, Institutional Review Board (IRB) and was determined to be exempt from the need for approval.

### 2.2. Immunohistochemistry

Immunohistochemical (IHC) detection of the various epitopes in human prostate cancer tissues was performed using the BenchMark ULTRA automated slide stainer (Ventana Medical Systems, Inc., Oro Valley, AZ, USA). All TMAs were stained using an anti-GLO1 polyclonal rabbit antibody (ab96032, Abcam, Cambridge, UK). At the same time, to discern normal prostatic tissue from prostate carcinoma, a dual standard stain [consisting of (i) a basal cell-directed antibody cocktail (34βE12+p63, VENTANA) and (ii) a rabbit monoclonal anti-p504s (racemase, SP116 clone, VENTANA), referred to as Rac/p63/HMWCK] was employed [34,35,36]. Sections from TMAs previously cut (4 µm) and stored were baked at 60 °C (60 min) prior to IHC staining. All steps thereon were performed on the VENTANA automated staining platform. Briefly, TMA sections were deparaffinized, pretreated with Cell Conditioning 1 for antigen retrieval, followed by inactivation of endogenous peroxidase. Specimens were incubated with anti-GLO1 rabbit polyclonal antibody (2 µg/mL) for 16 min at 37 °C. Immunoreactions were visualized using the OptiView DAB IHC Detection Kit (Ventana Medical Systems, Inc.). Following the chromogenic detection, all slides were counterstained with Hematoxylin II and Bluing Reagent (Ventana Medical Systems, Inc.) for 4 min each and coverslips were applied. System-level controls from cell line-derived FFPE included CAPAN-2, BT-474 and U-937 for weak, moderate and strong GLO1 expression, respectively. In addition, breast cancer tissue was used to monitor unspecific background staining on stromal cells. As a negative control, staining was performed in the absence of primary antibody. All reagents and incubation times on the dual staining assay were used as directed by the manufacturer. Although cytoplasmic and nuclear GLO1 staining was observable, only cytoplasmic staining was scored for biomarker expression. Intensity and prevalence of staining were assessed by a board-certified pathologist, and histologic scores (H-score) were calculated for each individual tissue core as previously described [37]. In the case of GLO1, complete absence of staining was considered ‘negative’, while an H-score of 1–100, 101–200, and 201–300 corresponded to ‘weak’, ‘moderate’ and ‘strong’ GLO1 expression, respectively.

### 2.3. Inclusion/Exclusion Criteria

Tissue cores were excluded based on the occurrence of staining artifacts (e.g., due to lack of reagent reaching the tissue or tissue cores detaching during the staining run). Additional exclusion criteria included tissue cores with folds due to poor adherence to the tissue slide. The final analysis included 797 tissue cores from 170 patient cases (Table 1). The majority of the cases had at least three evaluable tissue cores (with <10% displaying two or less evaluable tissue cores). Staining intensity and percent positive staining were scored for different tissue histologies (adjacent normal, HGPIN, PCa) and the calculated H-score was used for final analysis.

### 2.4. Statistical Analysis

GraphPad Prism 9.1.0 software (GraphPad Software, San Diego, CA, USA) was used for statistical analysis. Tissue data sets were analyzed employing non-parametric analysis using the Mann–Whitney test or the Kruskal–Wallis test (performed to evaluate associations between Glo1 expression and clinical variables); * *p* < 0.05; *** *p* < 0.0005; **** *p* < 0.0001.

## 3. Results

### 3.1. GLO1 Immunodetection during Tumorigenic Progression in Arrayed Prostate Patient Specimens

**Figure 1:**
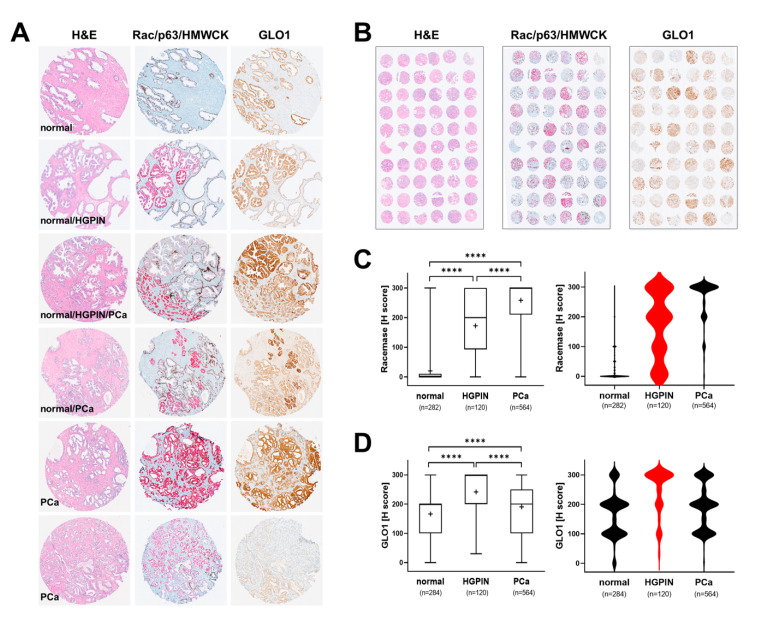
Comparative immunohistochemical analysis in prostate cancer tissue specimens: GLO1 versus standard (Rac/p63/HMWCK; ‘racemase’) staining. (**A**) Representative cores: H&E (left column), Rac/p63/HMWCK (middle column) and GLO1 (right column). (**B**) Representative TMAs stained as in panel A. (**C**) Racemase H-score comparison between adjacent normal, HGPIN, and PCa tissues (left: box and whisker plot; right: violin plot). (**D**) GLO1 H-score comparison between adjacent normal, HGPIN, and PCa tissues (left: box and whisker plot; right: violin plot). Statistical analysis of data was performed using the Kruskal–Wallis test (**** *p* < 0.0001); ‘+’ signifies the mean; HGPIN data depicted in red.

In order to comprehensively profile GLO1 expression during tumorigenic progression, tissue specimens originating from PCa (stage 2 and stage 3) patients were analyzed in TMA format (Figure 1A,B). For comparison, tissue was also analyzed using H&E and Rac/p63/HMWCK staining, performed according to established clinical standard procedures. Overall, as expected, racemase staining was highly elevated as a function of clinical occurrence of prostate adenocarcinoma and almost absent from normal tissue. Moreover, racemase was upregulated in HGPIN lesions as compared to normal control tissue and displaying a moderate staining intensity as compared to PCa (Figure 1A,C). A broad distribution of staining intensities detectable in HGPIN lesions was visualized by violin plot depiction, whereas the same analysis revealed a uniform expression characteristic of PCa specimens (Figure 1C, right panel). Remarkably, GLO1 was upregulated in HGPIN lesions, displaying a higher staining intensity (uniform among tissue specimens) than PCa lesions, as visualized by violin plot analysis (Figure 1D). Specifically, all HGPIN-positive cores (120/120) demonstrated positive GLO1 staining and 79/120 (65.8%) displayed strong GLO1 staining (Table 2).

Remarkably, out of 564 PCa cores, most displayed weak to moderate levels of GLO1 expression [weak: 152 (27%); moderate: 240 (42.6%)] and almost one-third of tissue cores demonstrated strong staining (169 cores; 30%) (Table 2). Taken together, these data indicate that whereas racemase expression increases linearly with tumorigenic progression, GLO1 expression is more characteristic of HGPIN lesions indicative of early progression.

Among these observations, pronounced GLO1 upregulation characteristic of HGPIN lesions is of particular interest given the role of HGPIN in PCa tumorigenesis. Morphological features of HGPIN as revealed by H&E staining include features such as a pronounced increase in nuclear size and chromatin as well as prominent nucleoli [7,8,38]. Indeed, the occurrence of HGPIN lesions is widely recognized as being indicative of carcinoma in situ with high predictive value of subsequent carcinogenesis, and HGPIN lesions are now considered being the most likely precursor of invasive PCa [7,8].

### 3.2. GLO1 Immunodetection in HGPIN as a Determinant of Adjacent Tissue GLO1 Expression

**Figure 2:**
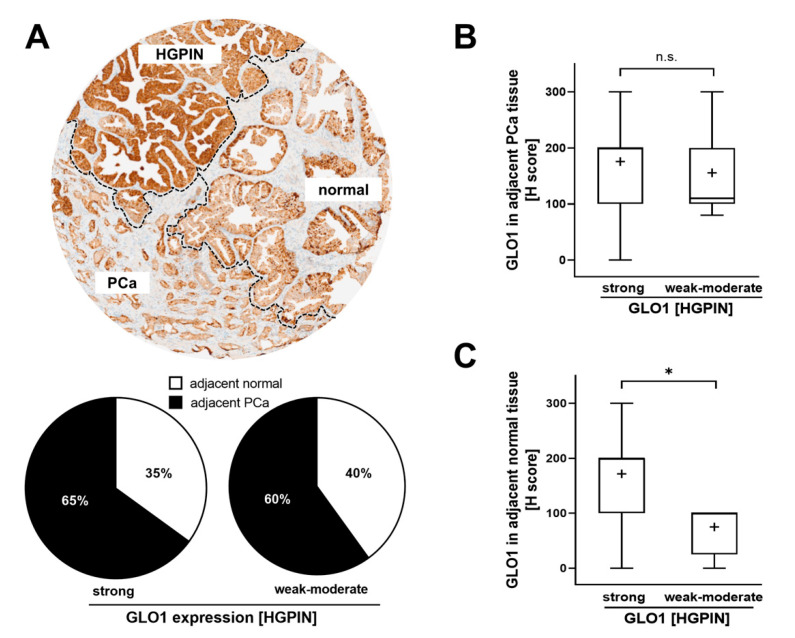
GLO1 immunohistochemical staining in HGPIN as a determinant of adjacent tissue GLO1 expression. (**A**) A representative core with HGPIN, adjacent normal, and PCa tissue (top panel). The pie chart (bottom panel) assesses the frequency (i.e., percentage of total cores scored) of normal and PCa tissue occurring adjacent to HGPIN [stratified by GLO1 expression status in HGPIN tissue; (strong GLO1: H-score 200–300; weak–moderate GLO1: H-score < 200)]. (**B**) GLO1 H-score in PCa adjacent to HGPIN with altered GLO1 expression (strong versus weak–moderate). (**C**) GLO1 H-score in normal tissue adjacent to HGPIN with altered GLO1 expression (strong versus weak–moderate). Statistical analysis was performed using the Mann–Whitney test (* *p* < 0.05); ‘+’ signifies the mean.

Next, we evaluated GLO1 expression in adjacent normal and PCa cores from cases with either strong or weak–moderate GLO1 immunostaining status detectable in HGPIN lesions. We also assessed the frequency of normal and PCa tissue adjacent to HGPIN. Although the number of HGPIN cores with weak–moderate expression of GLO1 was considerably lower to those displaying strong GLO1 staining, the proportion of PCa in both categories remained the same (60% vs. 65%) (Figure 2A). Of note, in the same tissue core, there were also cases displaying all three histologies (normal, HGPIN, PCa) adjacent to each other (see Figure 2A, top image). Importantly, PCa tissue adjacent to HGPIN displayed a similar H-score for GLO1 expression, independent of GLO1 expression status (weak–moderate or strong) observed in HGPIN tissue (Figure 2B). In contrast, normal tissue adjacent to HGPIN characterized by strong GLO1 expression exhibited high GLO1 expression, while GLO1 expression of normal tissue adjacent to HGPIN expressing weak–moderate GLO1 showed a significantly lower H-score (Figure 2C). Due to the rare occurrence of HGPIN characterized by weak GLO1 expression (4/42) statistical analysis was limited by the small sample size of low GLO1 expressing HGPIN. However, strikingly, analysis of a limited number of these specimens available to us revealed a clear trend suggesting a positive correlation between upregulation of GLO1 in normal tissue adjacent to HGPIN lesions displaying strong GLO1 expression (Figure 2C). Taken together, these data indicate that GLO1 status detectable in HGPIN correlates with GLO1 expression in adjacent normal tissue, a relationship not observable in PCa.

### 3.3. Association of GLO1 Expression with Gleason Grade, Pathological Stage, and BCR

**Figure 3:**
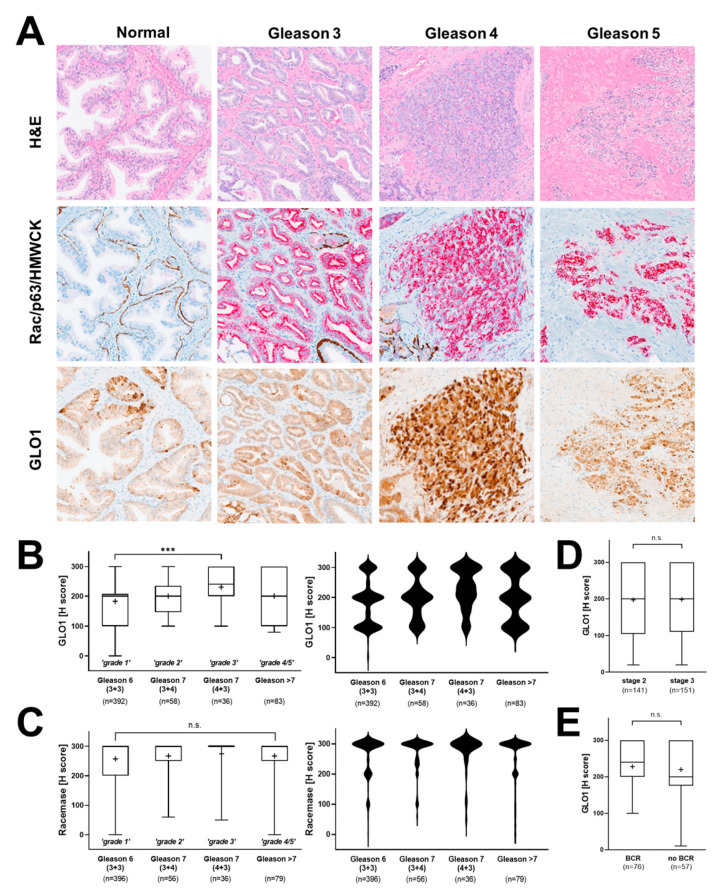
Association of GLO1 immunohistochemical staining with clinical parameters of PCa. (**A**) Representative images depicting different Gleason patterns from the tissue cohort; H&E (left column); Rac/p63/HMWCK (middle column); GLO1 (right column). (**B**) GLO1 expression as a function of Gleason grade (left: box and whisker plot; right: violin plot). (**C**) Racemase detection as a function of Gleason grade (left: box and whisker plot; right: violin plot). (**D**) GLO1 immunohistochemical detection as a function of pathologic stages 2 (organ confined) and 3 (capsular penetration). (**E**) GLO1 detection as a function of biochemical recurrence (BCR). Statistical analysis of tissue data sets was performed using the Mann–Whitney and Kruskal–Wallis tests (*** *p* < 0.0005); ‘+’ signifies the mean.

Next, we examined the potential association of GLO1 expression with clinical patient parameters. First, a potential relationship between Gleason grade and GLO1 expression status was examined (Figure 3A). Indeed, from Gleason 6 [3+3 (‘grade 1’)] to Gleason 7 [3+4 (‘grade 2’); 4+3 (‘grade 3’)], a significant increase in GLO1 H-score was observed. Violin plot analysis revealed that the frequency of highest GLO1 expression (H-score = 300) was observed in Gleason 7 (4+3), clinically referred to as ‘intermediate–high risk’ (Figure 3B, right panel). Gleason 7 (3+4), clinically referred to as ‘intermediate–low risk’ displayed diminished GLO1 expression as compared to Gleason 7 (4+3) yet elevated as compared to Gleason 6 (3+3) clinically referred to as ‘low risk’. Remarkably, GLO1 expression in tissue displaying Gleason > 7 (‘grade 4/5’) was not statistically different from Gleason 6 and was characterized by a broad range of GLO1 expression (Figure 3B). In contrast, racemase immunodetection of the same tissue cores revealed high (H-score > 200) expression levels irrespective of Gleason score, and violin frequency analysis indicated the uniformity of this expression pattern (H-score = 300) (Figure 3C). In contrast to the remarkable correlation between GLO1 expression and Gleason grade, patient-associated parameters [including pathological stage and biochemical recurrence (BCR)] did not display a statistically significant difference as a function of tissue GLO1 expression levels (Figure 3D,E).

## 4. Discussion

PCa is a progressive malignant disease with poor treatment outcomes when detected at late stages. Even though highly informative molecular markers exist that allow clinical detection of advanced malignancy (for example racemase- and cytokeratin-based markers), there is an urgent need for improved molecular biomarkers with diagnostic value that will allow detection of early-stage disease. Indeed, the availability of a valid biomarker indicative of precancerous stages would be expected to change current intervention paradigms that interfere with progression of PCa. 

Here, we show for the first time that pronounced GLO1 immunostaining is a distinct characteristic of HGPIN lesions analyzed in representative stage 2 and stage 3 prostate tissue specimens embedded in TMAs (Figure 1). Furthermore, GLO1 expression in HGPIN correlated with GLO1 expression in adjacent normal tissue (Figure 2). In addition, it was observed that GLO1 expression occurred as a function of Gleason grade up to 7 (‘intermediate–high risk’) with high statistical significance (Figure 3). Strikingly, we found that GLO1 upregulation is a consistent and specific molecular characteristic of HGPIN (precursor lesion to invasive PCa), and it is differentially expressed compared to PCa unlike the widely accepted biomarker, racemase with expression indistinguishable between HGPIN and PCa [34]. Thus, since current pathological assessment of HGPIN status solely depends on morphological features, our data suggest that GLO1 after further molecular and clinical validation may have utility as a novel diagnostic marker.

GLO1 upregulation in HGPIN lesions is of particular interest given the clinical role of HGPIN, widely considered as a premalignant state preceding PCa characterized by morphological features such as marked increase in nuclear size and chromatin, prominent nucleoli, and genomic instability [7,8,38]. Cumulative research supports the hypothesis that HGPIN represents early carcinoma in situ with high likelihood of this premalignant lesion progressing towards invasive PCa, a premalignant condition serving a comparable role as observed in other premalignancies such as hyperplastic polyposis in colorectal cancer (CRC), actinic keratosis in squamous cell carcinoma (SCC) and dysplastic nevi in melanoma [8,39,40,41]. Indeed, genomic analysis indicates that HGPIN and PCa share common genetic alterations [42]. Interestingly, upregulated GLO1 expression is a molecular characteristic observable even during later stages of PCa progression as documented by us as a function of Gleason grades 1 through 3 (‘low risk’ to ‘intermediate–high risk’) (Figure 3). Nevertheless, in contrast to the uniformity of elevated GLO1 expression in HGPIN, PCa specimens were characterized by a wide range of GLO1 expression levels, an observation with particular relevance to high Gleason grades (grade 4/5) (Figure 3).

Reprogramming of energy metabolism has now been identified as a hallmark of tumorigenic progression, and changes in GLO1 expression observable in precancerous and cancerous lesions of PCa patients must be interpreted in this context [31,43,44,45]. Strikingly, our observations indicate that advanced PCa specimens display a wide range of GLO1 immunoreactivity. It may therefore be hypothesized that metabolic adaptations associated with GLO1 expression serve an essential oncometabolic role during early stages of tumorigenesis (HGPIN), yet become dispensable at later stages of tumorigenic progression (Figure 1). Indeed, association of GLO1 expression observed in low to intermediate Gleason grade suggests that this occurrence represents an early oncometabolic switch relevant to PCa that becomes non-essential during later progressional stages of the disease. 

Additionally, it has been shown that even though normal prostatic epithelial cells rely on aerobic glycolysis (‘Warburg effect’) for energy production, reversion to mitochondrial OXPHOS-based energy metabolism during late stages of PCa progression has been documented [15]. Moreover, metabolic reprogramming in cancer cells might also occur in the context of crosstalk with the surrounding stroma and tumor microenvironment [46,47]. Thus, it is no surprise that normal tissue (adjacent to HGPIN lesions overexpressing GLO1) displays high expression of GLO1 consistent with the occurrence of molecular crosstalk and stromal support of malignant cells (Figure 2). In order to resolve these relevant questions, we are currently pursuing an experimental approach involving CRISPR/Cas9-based genetic target elimination that we have implemented successfully to explore the role of GLO1 in cancer cell lines including A375 malignancies melanoma and DU-145 PCa cells [21,44,45]. Concordantly, a recent publication has provided strong evidence that genetic modulation of GLO1 expression by siRNA impairs metastasis of PCa cell lines (DU-145, PC3). It was proposed that GLO1 antagonism, through elevation of cellular MG levels, causes TGFβ inactivation with suppression of TGF-β1/Smad EMT upstream of mesenchymal markers including vimentin, N-cadherin, MMP2, and MMP9 [31]. The availability of potent small-molecule drug-like GLO1 inhibitors currently used as pre-clinical therapeutics might also provide a powerful tool supporting mechanistic and clinical studies targeting GLO1 in PCa animal models and patients [48]. 

In summary, these data suggest that GLO1 may serve as a novel diagnostic biomarker of early PCa progression. Future research with inclusion of additional molecular parameters will substantiate the validity of GLO1 expression that selectively identifies HGPIN lesions in clinical samples and will also explore the mechanistic role of GLO1 as a causative effector in PCa progression that might also serve as a novel target for chemotherapeutic intervention at the early stage of the disease. 

## 5. Conclusions

In conclusion, we have identified GLO1 as a distinct molecular marker characteristic of early PCa development, as substantiated by high expression levels observable in HGPIN. Given its crucial role in regulation of glycolytic energy metabolism, GLO1 association with low to intermediate Gleason grade may be indicative of an early oncometabolic adaptation in PCa. Taken together, our data suggest that GLO1 may serve as a novel diagnostic marker for HGPIN detection during early PCa progression. Our ongoing research aims at substantiating the biomarker role of GLO1 that might also serve as a novel molecular target in PCa tumorigenesis.

## Figures and Tables

**Table 1:** Characteristics of the arrayed prostate cancer tumors and clinical data.

Characteristics	Total	%
No. of cases	187	100
No. unique cases w/evaluable cores	170	90.9
No. of cores	882	100
No. of evaluable cores	797	90.4
Age (years)	41–79 years old	
41–49	6	3.5
50–59	44	25.9
60–69	76	44.8
70–79	39	22.9
Unknown	5	2.9
Mean	63.4	
Pathologic stage		
2a	4	2.4
2b	2	1.2
2c	47	28.3
3a	109	65.7
3b	4	2.4
Unknown	4	2.4
PSA (pre-radical prostatectomy)	165 cases	
Unknown	5 cases	
Range	1–120 ng/mL	
Mean	11.56 ng/mL	
PSA (post-radical prostatectomy)	136 cases	
Unknown	34 cases	
Range	0–163 ng/mL	
Mean	2.43 ng/mL	
Biochemical recurrence	≥0.2 ng/mL (77 cases)	

**Table 2:** GLO1 immunodetection during tumorigenic progression in arrayed prostate patient specimens.

Staining Scores ^1^	Adjacent Normal (*n* = 286)	HGPIN (*n* = 120)	Tumor (*n* = 569)
Negative	12 (4.2%)	0 (0%)	3 (0.5%)
Weak	100 (35.2%)	18 (15%)	152 (27%)
Moderate	127 (44.7%)	23 (19.2%)	240 (42.6%)
Strong	45 (15.8%)	79 (65.8%)	169 (30%)
Total	284	120	564

^1^ H-score: weak (1–100); moderate (101–200); strong (201–300).

## Data Availability

Data presented is contained within the article; for additional information, data sets are also available upon request from the corresponding author.
